# Experimental Researches Concerning Febrile Caloricity, Both before and after Death—Post-mortem Fever

**Published:** 1844-10

**Authors:** Bennet Dowler

**Affiliations:** New Orleans


### WESTERN JOURNAL

OF

### MEDICINE AND SURGERY.

---

OCTOBER, 1844.

---

ART. I.—*Experimental Researches concerning Febrile Caloricity, both before and after death—Post-mortem Fever.* By BENNET DOWLER, M.D., of New Orleans.

(CONCLUDED.)

In order to give this paper the greatest possible authenticity, I drew up a detailed account of all my facts, together with the manner and order of proceeding; these I abridged, but still they assumed a form too massive for a monthly journal; and now I am compelled to give results, rather than processes. This I regret the more, because I am unable fully to develope the most interesting feature in this inquiry; viz:

the regional caloricity at different periods, especially at different periods in the dead body.

All authors, so far as recollected, represent the human body after death as cooling, like inert matter, according to a physical law discovered by Sir Isaac Newton, and well expressed by Pictet from whose work I translate the following concise statement:

"For a long time it was thought that this law was exact; but when people wished to verify it, they found that it was only true in cases where the temperature of the body did not exceed that of the surrounding air more than from 45° to 50°," (113° to 122° Fah. covering the whole ground of my observations): "for still greater differences the law is inexact, and the more so as the difference of temperature is considerable.

"When a solid body of what form soever cools itself in a medium of constant temperature, it is evident that the temperature of the body ought to decrease from the surface to the interior, but that the differences of temperature will cease as the refrigeration shall progress, and that the temperature of all the points of the mass must terminate by becoming uniform, and equal to the surrounding medium, in a longer or shorter time: it is then only that refrigeration will be complete. M. Fourier has determined all the circumstances of the refrigeration and heating of solid bodies by setting out with the hypothesis that the radiation of one molecule was proportional to the difference between its temperature and those of the surrounding molecules."

Beclard says:

"The warmth of the body sometimes diminishes before death, and ceases altogether soon after. The cooling takes place gradually, and *commences at the surfaces and extremities.*"

This law, so far from applying, is reversed soon after death, in this city at least, in bodies recently dead from fever, with, perhaps, a very few exceptions, as will appear hereafter.

THE VELOCITY OF HEATING MERCURIAL THERMOMETERS IN THE HAND.

The bulb of a cold thermometer when taken in the hand, or under the arm, robs the part of its heat, or produces a local refrigeration which cannot be fully replaced, until the heat of the whole mass shall re-establish the equilibrium, which, I am inclined to think, requires from ten to fifteen minutes, in most cases where great precision is desirable, though for practical purposes a period less prolonged may be sufficient. On nine consecutive days, beginning April 15th, the following results were obtained with Reaumur and Fahrenheit's thermometers, amounting to 108 observations with each. The experiments lasted about fifteen minutes each, and were made upon the same person.

The Reaumur instrument here used, the one with which I made nearly all my experiments, was, after repeated trials, found to be only half a degree too high when subjected to the test of melting ice, a rare accuracy in these instruments, according to my experience. Its boiling point was exact. This thermometer offers during the first five minutes in the morning a discrepancy. I made no observation on the sick or dead with it.

| | 9 A.M. | | | M. | | |
|---|---|---|---|---|---|---|
| THER. | 1st five minutes. | 2nd five minutes. | 3rd five minutes. | 1st five minutes. | 2nd five minutes. | 3rd five minutes. |
| REAU. | 90.66° | 96° | 97.55° | 95.77° | 99° | 99.94° |
| FAH. | 92.6° | 94.72° | 96° | 96.88° | 97.83° | 98.66° |

| | 3 P.M. | | | 9 P.M. | | |
|---|---|---|---|---|---|---|
| THER. | 1st five minutes. | 2nd five minutes. | 3rd five minutes. | 1st five minutes. | 2nd five minutes. | 3rd five minutes. |
| REAU. | 94.38° | 97.88° | 99.61° | 94° | 98.05° | 99.33° |
| FAH. | 95.33° | 96.75° | 98.33° | 96.94° | 98.26° | 98.5° |

THE VELOCITY OF COOLING IN FAHRENHEIT'S THERMOMETER, AFTER BEING HEATED WITH ANIMAL CALORIC.

These observations were made on nine consecutive days, ending April 23d. The weather was clear, and gave an average temperature of 70.55° at 8 A.M., of 81.44° at 2 P.M., of 71.5° at 8 P.M. The instrument was heated, perhaps not always to the minimum of the hand, and gave 96, nearly, as its average point of departure; the mean fall of the quicksilver at the end of every five minutes was as follows: 83.94° 79.12°, 78.22°, 77.57° 77.25°.

In a case of chronic pneumonia, the patient was bled one pound, in an open gallery, the air of which was 83°; the blood falling on the thermometer raised the mercury to 94°, but it began to fall as soon as the blood ceased to flow; in 12 minutes it fell to 92°, in 10 to 90°, in 15 to 87°, in 25 to 83°, in 30 to 81°, in 15 to 79°. The bowl of blood reheated artificially to 95°, being placed in the same situation, the air being the same, the mercury fell in 12 minutes to 86°—in 12 to 83½°, in 15 to 81°, in 15 to 79°, the stationary point, which was reached in about one *half the time required to dissipate the animal* caloric.

Next, a similar quantity of rain-water was treated as in the last experiment, and gave within one degree the same ratio of cooling. In acute syphilis, a pound of blood just drawn gave 92°; in 12 minutes after 84°, in one hour 67°, the air being 62°. In a case of yellow fever, the blood raised the mercury in two minutes to 98°, at which it remained stationary two minutes, and then began to fall; in another case, the temperature of the blood was 100°, as long as it was observed, that is, for 10 minutes. These facts though few, and perhaps inconclusive of themselves, assume a more important character when connected with many more decisive experiments to be mentioned hereafter, indicating *that morbid caloric differs from other kinds of caloric of the same temperature.*

THE EQUAL DIFFUSION OF PHYSIOLOGICAL CALORIC IN THE BODY.

Without suspecting any one of bad faith, it is easy to see that in many things relating to temperature, one author copies another, instead of entering upon the verification of his facts. Thus all authors, so far as I know, represent the temperature of all parts of the healthy body as constantly diminishing in proportion to their distances from the heart or centre. This is certainly a mistake in the recently dead body, as my numerous observations prove beyond question, when the death is caused by fever; though in congestive diseases it is no doubt true before death.

Without alluding to the analogical proof drawn from the dead body, and from some other sources, I offer the following summary of several hundred observations made on one person, in bed, with scarcely any interruption during 11 days, comprehending the close of April and the beginning of May, under circumstances as similar as could be conceived, plainly showing that from 4 to 6 o'clock, A.M., the caloricity of the body at every accessible point, is exactly alike, if we except a minute fraction of a degree in the popliteal region, owing, perhaps, to the inconvenient position, not allowing the observer to make the temperature accurately give the mean temperatures:

Room 74°—hand 98.25°, axilla 98.4°, mouth 98.33°, perineum 98.3°, popliteal region 97.8°, sole of the foot and inner ankle 98.47°. At 9 o'clock, A.M., after rising from bed, hand 96.5°; at noon and at 9 P.M. 99°. Mouth at 9 A.M. and at M. 99°; axilla at 9 A.M. nearly 99°; sole and ankle at different periods of the day some hours after rising, 94°. The maximum of the air during these 11 days was 87°—its minimum 68°. This falling of the temperature of the feet, after rising from bed, so far from admitting of explanation upon the principles of the physiologists, plainly contradicts their theory, which ascribes the diminished heat in the extremities to a diminished amount of blood in remote parts, for on getting out of bed it will be seen, that the blood from its gravity rushes into the legs and feet distending the vessels instantly

1 *

and ought, according to their doctrine, to make the parts warmer. Again, they declare that when an artery which supplies a part with blood is tied, 'heat is no longer disengaged from it.' The agony rather than the diminished supply of blood may at first produce such a result. A young, vigorous man received a slight stab in the axilla with a pen-knife, which punctured the axillary artery, causing a small elongated aneurism, for which Dr. Mercier, of New Orleans, tied the subclavian artery, on the 2d day of November, 1843. The arm was soon diminished in size, and at death, on the 18th, was greatly withered, and much smaller than the other. About 36 hours after this formidable operation, the arm was without arterial pulsations, but gave exactly the same temperature as that of the unaffected arm: both raised the mercury to 102°, the patient being rather feverish at the time.

The summary drawn from the following averages, carefully prepared from accurate tables during 16 continuous days of the month of June, shows that in a healthy person the temperature of the hand and of the urine is the same—a fact not only proving the physiological dogma which claims for the centre a heat much greater than that enjoyed by the extremities, to be erroneous; but it suggests an easy and direct route by which to ascertain the morbid heat of one great centre during life without vivisections, or the equivocal method of acupuncture, adopted by Becquerel and Brechet.

During the period above mentioned the air averaged in the mornings and evenings at the time of the observations, 83.2°, the hand 99.5°, and the urine 98.37°. There the hand was 1.13° hotter than the urine; but as more or less heat was necessarily absorbed by the glass vessel receiving the urine, notwithstanding every precaution, it may be fairly assumed, that the palmar and pelvic regions are equal in temperature.

ANTE-MORTEM SERIES. ANALYSES OF SUMMARIES.

*Analysis of table No.* 1, *showing the primary period of yellow fever in persons that recovered:*—24 cases: mean time of illness when the observations were taken, 26.81 hours; mean of air 81.3°; mean of the hand 101.22°; maximum 107°; minimum 95°; mean of the axilla 103.11°; maximum 109°; minimum 102°.

*Analysis of table No.* 2, *showing the temperature of yellow fever in the middle periods, in those who recovered:*—40 cases: mean time of illness when the observations were made 5.92 days; mean of the air 82.52°; mean of the hand 100.42°; maximum 107°; minimum 94°; mean of the axilla 203.11°; maximum 109°; minimum 97°.

*Analysis of table No.* 3, *showing the temperature of the convalescent period of yellow fever patients:*—26 cases: mean time of illness when the observations were made 6.50 days; mean of the air 82°; mean of the hand 96°; maximum 100°; minimum 91°; mean of the axilla 98.46°; maximum 102°; minimum 83°.

*Analysis of table No.* 4, *showing the temperature of the primary period of those who died of yellow fever:*—12 cases: mean time of illness when the observations were made 24.62 hours; mean of the air 83.75°; mean of the hand 102.54°; maximum 197°; minimum 97°; mean of axilla 105.91°; maximum 109°; minimum 100°.

*Analysis of table No.* 5, *showing the temperature of the middle period of those who died:*—25 cases; mean time of illness when the observations were made 6.04 days; mean of the air 81.16°; mean of the hand 99.4°; maximum 106.5°; minimum 91°; mean of the axilla 103.39°; maximum 107°; minimum 99°.

*Analysis of table No.* 6, *showing the temperature of the fatal stage of yellow fever:*—51 cases: mean time before death when the observations were made 16.01 hours; mean of the air 81.24°; mean of the hand 92.25°; maximum 104°; minimum 81°; mean of the axilla 99.56°; maximum 106.5°; minimum 90°.

POST-MORTEM SERIES. ANALYSES OF SUMMARIES.

*Analysis of table No.* 1, *showing the post-mortem fever of regions:*—43 dead bodies: mean time after death, when the observations began 29.5 minutes; maximum delay in three cases 3 hours; minimum 1 minute; mean duration of the experiments 1 hour and 32 minutes; 4 maxima, being, 1 of 4 hours and 8 minutes, and 3 of 4 hours' duration: mean of the air 84.4°, partly taken in the dead-house, and the residue from other records; mean of the axilla 104.44°; maximum 109°; minimum 96°; mean of the thigh 104.71°; maximum 113°; minimum 100°; mean of the rectum 104.05°; maximum 111°; minimum 100°; mean of the epigastrium 105.48; maximum 111°; minimum 101°; mean of the chest 102.95°; maximum 107°; minimum 97°; mean of the heart, generally of the right side, 103.5°; mean of the brain 98.71°; maximum 102°; minimum 95°. The chest and rectum are omitted in about half the cases, and the brain, perineum, pelvis, liver, and heart, in a proportion somewhat greater. The liver was observed in nine cases, and gave a mean of 106.33°; a maximum of 112°, and a minimum of 102°. The perineum without incisions gave a mean of 104.45°; a maximum of 109°; and a minimum of 101°, in 10 cases. The pelvis and lower abdomen in 9 cases averaged 105.05°; maximum 107; minimum 100°.

This table is sufficiently curious, but we utterly despair of showing from it by a comparison of organs at different periods, the surprising phenomena manifested by *post-mortem* fever, in particular regions remote from the centre, at prolonged intervals after death, requiring hours to reach its maximum; then, sometimes declining to the temperature of the centre, both keeping pace for a time, and then the centre falling more rapidly, leaving the thigh stationary, at perhaps. 106°, for many minutes—reversing all the laws of refrigeration known to philosophers. So we leave this subject for the present.

Owing to circumstances not necessary to mention, it often happened that the *post-mortem* fever had begun to decline—

actual refrigeration had progressed upon the surface—before I commenced the observations. In selecting bodies, my preference was for those the most recently dead. When a body begins to refrigerate, of course the parts most distant from the centre will cool first. In this way the average of the thigh, great as it is, is more or less reduced, but I have not for that reason suppressed a single case. It will be seen that the thigh affords a maximum of 113°, exceeding every other region. Let us compare five maxima of the eight principal regions:

| Thigh. | Epigast'm. | Axilla. | Chest. | Heart. | Brain. | Rectum | Liver. |
|---|---|---|---|---|---|---|---|
| 113° | 111° | 109° | 107° | 109° | 102° | 111° | 112° |
| 109° | 110° | 109° | 106.5° | 106° | 101° | 109° | 109° |
| 109° | 109° | 108° | 106° | 105° | 101° | 107° | 108° |
| 109° | 109° | 108° | 106° | 104° | 100° | 107° | 107° |
| 108° | 109° | 107° | 105° | 104° | 99° | 106° | 106° |
| M. 109.4° | 109.6° | 108.2° | 106.1° | 105.6° | 100.6° | 108° | 108.4° |

*Analysis of table No. 2, showing the decline of post-mortem fever, or the incipient refrigeration of regions, the forerunner of putrefaction:*—17 cases: mean time dead when the observations began 6 hours and 48 minutes; mean of the air about 86°; mean of the thigh 94.26°; maximum 103°; minimum 84°; mean of the epigastrium 97.5°; maximum 106°; minimum 84°. The following regions were not examined in all the dead bodies: mean of the lungs 94.28°, of the heart 93.25°, of the liver 96.5°, of the rectum 99°, of the axilla 96.88°, and of the brain 85.33°.

*Analysis of table No. 3, showing the period of refrigeration* in which the body and surrounding media coincide, very nearly in temperature, and in which putrefaction declares itself, accompanied by suppleness of the limbs, softness of the muscles, relaxation of the cornea, abdominal distention, green discolorations of the skin, and fetid gases:—19 cases: mean time dead 17.04 hours; mean temperature of room 84.39°; mean of the thigh 85.35°, of the epigastrium 88.11°, of the lungs 89°, of the axilla 86°, and of the brain 79.12°.

The common opinion that putrefaction is accompanied or caused by an augmented heat in the human body, is quite erroneous, as we shall see hereafter.

Since it is quite impossible, on the present occasion, to relate the details in more than two hundred histories of temperature, I will attempt to give you a faint outline of several, omitting all minor matters.

History 1st.—Highest temperature during life in the axilla, 104°; 10 minutes after death axilla 109°; in 15 minutes the thigh gives 113°, 9 above the living maximum; in 20 minutes the liver gives 112°; in 1 hour 40 minutes heart 109; thigh, old incision, 109°; in 3 hours after removing all the viscera, a new incision in the thigh gave 110°, 6° above the living maximum.

Hist. 2d.—Alive: axilla 100°, hand 91°; dead 1 hour and 5 minutes: axilla, at the end of every 5 minutes, 102°, 104°, 107°, thigh 107°; 2 hours after death centre of the left lung 106°, apex 104°, heart 104°, thigh 106°; 3 hours, axilla 104°, liver 106°, thigh 106°—repeated, 106°, rectum 105°; 23 hours after death, room 90°, thigh 88°; putrefaction developed.

Hist. 3d.—Last stage, hand 91°, axilla 100°; dead 30 minutes, axilla 104°; perineum, without incision, 102°; rectum 102°; epigastrium 103°; brain, through the orbit, 102°; body appeared to be growing hotter, when demanded by friends for interment.

Hist. 4th.—Last stage, hand 70°, (7° less than the air); axilla 95°; about 2 hours after death, axilla 100°, rectum 104°; axilla rose to 102°, epigastrium 101°, thigh 102°, brain 99°, heart and left chest 100°.

Hist. 5th.—2 days before death, hand 101°, axilla 104°; 1 day before death, hand 100°, axilla 100°; dead 5 minutes, axilla gave at different periods, and was still rising, 103°–4°–

5°–6°, epigastrium 106°, brain 101° and falling; the thigh 101°, though exposed to a cold wind, which sunk the mercury 10° during the observations.

Hist. 6th.—4½ hours after death, thigh exceeded the brain 6°, the chest 3°.

Hist. 7th.—Air cold, (Oct. 26), mercury falling 3 or 4° per hour: dead 1 hour, axilla 103°, epigastrium 104°, nearly, liver 108° and rising, brain 88° falling; 2 hours after death, thigh 104°, left chest 97°.

It is confidently believed, that these outlines of a few cases, taken almost at random, will serve to illustrate the principal features which belong to the whole. I wish rather to avoid the extended history of morbid temperature, in the living body, though here my materials are most ample, in order to give the more attention to its *post-mortem* phenomena.

These facts, and they are but a few of those I have collected, show most conclusively, that the febrile subject does not, as is universally supposed, fall under the dominion of the Newtonian law of refrigeration at death, for a period, often, greatly prolonged. Even when refrigeration commences it seldom follows this law rigidly, at first, as it does not proceed on the surface, in a ratio proportioned to its distance from the centre, and in any arithmetical progression, at each successive period of time. The calorific maximum is often found at the surface; then it is, perhaps, set up in the centre, soon to reappear on the circumference. The body, like a metallic statue, will then discharge equal degrees of heat, in equal intervals of time, until an equilibrium is established between it and the surrounding media, as before explained. Does not this afford presumptive proof, that morbid caloric differing as it does in laws, must differ in nature and origin from common caloric derived from the sun, the earth's centre, the consolidation of bodies, and friction?

This morbid caloricity, so far from causing, appears to retard, putrefaction. All our facts prove that this must first be dissipated before putrefaction begins; then the air and body coincide, and, if the former be not too cool, decomposition declares itself. I must beg the reader to call to mind what Baron Larrey says of the effects of cold upon the French army in the retreat from Russia in 1812. "When they came near the fire," says he, "instant gangrene took place; its progress was perceptible to the eye." "The Russians know that a fish thawed in warm water will become putrid in a few minutes" (Vide Mem.) I am told by house-keepers, that butcher's meat kept in ice the night before sale, often becomes putrid before meal-time. In a case of compound fracture of the right leg proving fatal on the third day, (December 13), air 54½°, the gangrened limb, black, fermenting, puffy, enormously distended, gave, in the thigh, which was not quite reached by the gangrene, nearly 5° less than the sound limb at the corresponding point, about three hours after death.

Physiological heat always exists at a degree favorable to to putrefaction, yet it proves antagonistic or conservative in its character; when, however, animal is replaced by a certain degree of physical heat, decomposition is developed; and this is a distinction, by no means idle or devoid of pathological interest.

Physical, as well as animal caloric, may undergo deleterious changes or degenerations, not perhaps appreciable by the mere laws of temperature. Of this character appears to be the wind called Harmattan or Sirocco, described as

> "Vapors blown by Auster's sultry breath,
> Pregnant with plagues and shedding seeds of death."

Mr. Wellsted, a late scientific traveler, says, that at Bagdad this wind kills all who are unsheltered from its influence; and Baron Larrey from experience and personal observation confirms the whole of Volney's frightful account of it: "the lungs and skin crisp; respiration becomes laborious; febrile heat, hæmorrhages from the nose and mouth, and suffocation take place: the dead bodies swell prodigiously and putrefy rapidly." Dr. Forry, the learned climatologist, quotes Dr. Jno. Davy's account of this wind, which, according to Dr. D. is never above 86°, in Malta, the Ionian Isles, and the Mediterranean. If caloric cause the Sirocco, it must undergo some deleterious change independent of this very common temperature, (86°) in all parts of the torrid and temperate zones.

The geography of solar heat, especially in African climates, presents a very interesting study in a pathological point of view. All exploring expeditions to that country have been almost completely annihilated. Mungo Park, in the Journal of his second Expedition to discover the termination of the Niger, says, that on the 10th day of June, 1805, in *three minutes* after the rain set in, many of the soldiers were affected with vomiting, others fell asleep, and seemed as if intoxicated; on the following day more than one-fourth were on the sick list, and on reaching the Niger only five persons out of forty-four, that left Gambia in perfect health, were alive. In these arid plains, marsh poisons could have no existence. Gibbon says that the generals of Augustus Cæsar "attempted the reduction of Ethiopia and Arabia Felix. They marched near one thousand miles to the south of the tropic, *but the heat of the climate soon repelled the invaders*, and protected the unwarlike natives of those sequestered regions."

But this suggestion is worthy of more consideration than can be given to it in this place, entering as it does, as the chief element into acclimation, a process which, in New Orleans at least, secures the constitution as completely against all grave fevers, as vaccination does against small pox, provided the person does not leave the charmed circle which forms the limits of the city. This process, so far as my personal observation goes, is almost peculiar to New Orleans.

Though I have the facts and statistics to prove that the natives and acclimated citizens are thus protected, yet, this may be regarded as one of Lemuel Gulliver's stories, so long as this city averages every year a loss of life probably as great as the annual loss from actual war, at any time in our country's history. I have witnessed epidemics in which nearly the same number fell as were killed in the battle of Waterloo under Wellington. In 1841, for example, 1,800 died of yellow fever: of these four were certified as having been born in New Orleans; two of these were aged 2 years each, probably born of unacclimated parents; one was 5 years old; the fourth 22. The two latter had probably lost their acclimation by absence.

In a fluctuating population of 40,000, it is most reasonable that three children and one young man should first have seen the light in New Orleans, without afterwards remaining constant residents. If we do not throw these out altogether as we ought, the mortality is nothing, compared to that of secondary small pox.

A line drawn from Washington City to Clarkesville, Virginia, and thence to St. Louis, will pass over a country much of which I know. There is not, so far as I have observed, one acclimating disease in that whole route; the *native* has no exemption from fevers, beyond the *stranger*. McCulloch, in his elaborate Statistics of the British Empire, shows that the mortality of the British army is nearly the same in the West Indies, the first 5 years as the second 5 years, being in the first period, annually, 10.4 out of every 100; during the latter nearly 10 per cent. per annum. By way of illustration he adds, "we can never accustom men to take arsenic." Yellow fever belongs to hot climates, and to hot seasons, though not to the hottest necessarily; mere elevation of temperature, or extreme heat does not indicate its approach; the hottest seasons are often healthy.

It is not unlikely, that in urban situations, as well as in the deserts of Arabia, solar heat may undergo deleterious changes, not appreciable by the thermometer.

It will be seen on comparing the results of the two tables representing the convalescent and dying stages, that in the latter, the axilla is hotter, while the hand is colder than in the former, but in neither is the temperature elevated as in the early stage. Now this coincidence between the dying and the convalescent, is *apparent*, perhaps, not *real*. There can be no reason to doubt that the whole mass of the body discharges its morbid heat in recovery: on the contrary it recedes from the surface of the dying, now bathed in the dews of death, and concentrates upon the centre. Death takes place. The lungs and skin cease to refrigerate. The true fever no longer modified or repressed soon returns with its smouldering fires, until the heated mass, at last antagonised by the surrounding agencies, and its peculiar heat being dissipated and replaced by mere physical heat, it falls under the domination of chemistry forever.

Never were experiments made with greater fairness. I might have omitted with justice not a few cases where, probably, the *post-mortem* fever had declined before I had the opportunity of making the observations; yet with all these cases, tending to lessen the average temperature, the thigh and axilla of the dead rise to nearly the mean of 105° each, more than 20° above the mean of the air, more than 12° beyond the mean of the hand in the last stage, almost exactly agreeing with the axillary mean in the first stage of the *ante-mortem* fever.

The place where the *post-mortem* fever was studied was well adapted to produce speedy refrigeration; nearly all of one side of the room was composed of lattice-worked doors; the wind had free access to the body, which was placed on a stone floor, the corpse had no clothes on, or other covering than a linen sheet which was generally removed.

No explanation of the *post-mortem* heat can be drawn from the coagulation of the blood, the rigidity of the body, etc., since the heat existed when they were absent, and the contrary.

It will be seen that the great nervous centre to which some ascribe animal heat, the brain, is in all cases the coolest part—the first to refrigerate, and the only one in which *post-mortem* fever never appears to any considerable extent, its maximum being 102°, less than that of the thigh by 11°; its mean 97.71°, exactly 6° less than that of the thigh. Now this is the more remarkable as the brain from its great size, and from its being surrounded by a non-conductor, hair, ought, *a priori*, to yield its heat by contact and radiation more slowly than any other organ.

These experiments are quite sufficient, if I may be allowed to judge, to overthrow the doctrine which ascribes the origin of animal heat solely to respiration. Take, for example, the following outline of a single case: dead 2 hours 30 minutes, experiments 3 hours 59 minutes: air 86°, axilla 109°, rectum 107°, perineum 109°, pelvis and abdomen 107°, liver 107°, epigastrium 105°, chest and heart each 102°. Virey says that the lungs constitute the only bed of the animal heat, yet, except the brain, this bed of heat is the coolest organ between the knee and the crown of the head, its maximum being 106½°, that is 6½° less than the thigh, and its mean 102.95°.

At the decline of the *post-mortem* fever, or at the period of incipient regional refrigeration, the thigh gives a mean of 94.-26°, the lungs a mean of 94.28°; at the period of incipient putrefaction, the mean of the former is 85.35°, that of the latter 89°. In but four cases, however soon after death, have I found the *post-mortem* fever as high in the lungs as in the thigh; while in many cases the latter was vastly hotter.

Haller, distinguished alike for common sense, and uncommon learning, asks, "Is the blood cooled in the lungs?" He says, "when the lungs are obstructed, ulcerated, almost destroyed, morbid heat is increased." And we know that this is true—we know how pungent is the heat of the consumptive. How could this happen if the heat was developed exactly in proportion to the size of the lungs, and the perfection of the respiration? In the language of Haller, "Is not the blood *cooled* in the lungs?" Are not the lungs a pair of refrigerators?

This paper fully demonstrates that a great developement of morbid heat takes place upon the surface, at the invasion of yellow fever, and though in the closing period, especially in the agony, a temporary recession occurs as to the surface, yet, no sooner is the conflict ended, than the original morbid caloricity, no longer rendered latent by evaporation from the skin and lungs, reappears in all its intensity for a period considerably prolonged. If we suppose the central, the great vital, organs to be as hot during life as they are found to be soon after death, the only wonder is that vitality should maintain its seat for a week or more, under the positive changes that ought, by every law of caloric, to take place in the molecular arrangement of the tissues. Let us suppose the brain in life to become as hot as the thigh is found to be after death, that is 14° or 15° above health; the cerebral mass would necessarily expand faster than its cranial walls; the fluids would dilate, and perhaps transude; compression would be the consequence, attended with convulsions, coma, and other effects incompatible with life. Suppose any other organ should become such a focus of morbid caloric only for a moment, would not each vessel from dilatation lose its healthy elasticity and cohesion, and thus pave the way to sanguineous congestion, emitting the blood to the part as effectually as the cupping glass does, when the pressure of the atmosphere is removed. In some local diseases, the lesions will afford an average alteration as great, if not greater, than fatal gun-shot wounds; as, for example, dysentery, consumption, and cancer. But in fever how much is entangled, and unexplained! Is not morbid caloric the agent that eludes the knife of the anatomist? To say nothing of its directly poisonous, let us consider its mechanical effects, as above mentioned, upon the brain. After dilating its delicate vessels, and establishing a sanguineous congestion, death follows. The brain, as we have shown, falls sooner than other parts under the law of refrigeration; the cranium contracts; this tremendous force drives the blood down from the brain towards the warmer and more yielding centres of the trunk; perhaps a real apoplexy, without rupture, has disappeared. The febrile subject offers many instances of great vascularaity of the vessels of the pia mater, without turgescency; the veins especially, are found full but flattened as if by pressure. There can be no doubt that in the living, as well as in the dead body, foci of caloricity establish themselves in particular parts, sending off, not always in right lines, but in deflected currents, morbid heat to certain organs, passing by others. Thus the epigastrium and axilla may stand charged with a positive caloricity of 109°, while the organs ot that part of the chest lying between these points shall be in a negative, or much lower state of temperature. I could muster serried columns of facts illustrative of some other points but I must omit them altogether.

So far as morbid caloricity can be identified as a cause cf disease, we deal with a positive, not an imaginary agent, where the ground is not eternally slipping from beneath our feet. Albumen, which abounds in the brain and fluids, coagulates at 160°; hematosine, the coloring matter of the blood, at 149°; and moderate increase of heat vastly augments the solvent powers of the serum over gelatine, so abundant in the body. The phosphorus in the body, were it uncombined, would burn in a heat less than 113°.

Admitting that the whole body be permeated with 10° or 15° of heat, and that it cannot render this heat latent, I ask, again, is it wonderful that death should ensue? Which atom has not undergone a deleterious modification, or a new arrangement in its chemical, mechanical, and vital laws and relations? "Delaroche and Berger prove that animals, in chambers heated to 120° or 130° Fah., have their temperature raised 11° to 16°, and die speedily." If, as some maintain, all lesions may be reduced to those of nutrition, caloric is an agent well adapted to play an important and fundamental part, not only diminishing the elementary adhesion of the tissues, but in debilitating all the organs, thereby favoring intertextural depositions, hypertrophies, softenings, hæmorrhagic and serous effusions, morbid secretions, engorgements, and other alterations, solid, liquid, and gaseous.

This whole subject is still in a very unsatisfactory state. I am very far from advocating the exclusive igneous pathology of fever. I have already explained the accidental origin of this laborious experimental inquiry, to which my attention has been directed for less than one year. It forms no part of my present design to enter upon the practical suggestions growing out of these researches. The imperfect state of pathology represents but too truly the condition of therapeutics. Were Æsculapius himself to revisit the earth, to ascertain the progress and present state of medicine, he could scarcely tell, without taking the votes of his disciples, whether excitants or antiphlogistics, quinine, morphia, camphor, and porter, or deliquium bloodletting, leeches, cups, iced lemonade, and gum-water; the cold water legions of the Silesian, or the incendiary army of red-pepper, alcohol, and hot steam, could muster the greatest force. The son of Apollo would find himself in the midst of a general *melé.* He would witness the collision of pride with pride, cupidity with cupidity, envy with envy, interest with interest—a shock that would cause the 'honest ghost' to sigh for the society of his illustrious medical companions of the Elysian fields, where fraternization prevails, and truth stands revealed, one and indivisible.

New Orleans, July, 1844.

